# App fatigue in mHealth: Beyond improving apps, advance equity by meeting people where they are

**DOI:** 10.1371/journal.pdig.0001107

**Published:** 2025-11-21

**Authors:** Shahmir H. Ali, Hein Thu

**Affiliations:** 1 Saw Swee Hock School of Public Health, National University of Singapore, Singapore, Singapore; 2 Digital Transformations for Health Lab, Geneva, Switzerland; National Tsing-Hua University: National Tsing Hua University, TAIWAN

## When more becomes less: The rising toll of app fatigue

The past decade has seen an extraordinary surge in digital health technologies, with more than 350,000 health applications (apps) now populating app stores. In 2020 alone, 90,000 were released—nearly 250 each day [1]. This proliferation reflects optimism that digital tools could extend healthcare reach, empower patients, and reduce barriers to care [2]. Yet most apps remain narrowly focused on basic tracking, offer limited functionality, and rarely undergo rigorous testing for effectiveness, safety, or equity impacts [3].

At the same time, this optimism has increasingly been tempered by recognition that the rapid expansion of fragmented and overlapping tools can overwhelm rather than enable users, a problem described as app overload [4]. App overload refers to the excessive number of digital health applications that create cluttered, inconsistent user experiences and require constant switching between platforms. Over time, this contributes to app fatigue [5], which describes the cumulative sense of burden, disengagement, and cognitive exhaustion that arises from managing too many disconnected tools. App fatigue reflects not only frustration with the number of available apps but also the ongoing effort of navigating, logging into, and maintaining multiple systems and data streams.

App development has outpaced evaluation mechanisms, leaving patients and clinicians overwhelmed by choice and uncertain about which tools to trust [4,6]. Platform fragmentation can discourage sustained use and care integration [4]. Poorly utilized apps lose their potential to improve outcomes, with disproportionate effects on those with lower digital literacy. Multiple apps generate overlapping notifications and information overload, which recent evidence links to anxiety and unnecessary health service use [7].

Importantly, app fatigue is often distinct from issues of app quality or access. While poor-quality apps fail to meet users’ needs and access barriers limit who can participate [8], app fatigue can emerge even when tools are well designed and affordable. It reflects a deeper structural problem of cognitive overload and ongoing management demands [9], where the effort required to navigate and coordinate multiple systems can erode users’ confidence in digital tools, disrupt continuity of use, and weaken long-term engagement. Although some empirical work has begun to examine the correlates and implications of app fatigue [5,7], the concept remains relatively new, and systematic evidence on its effects on trust, engagement, and health outcomes is still limited [8].

Moreover, the challenges associated with app fatigue may be even more pronounced in low- and middle-income countries, where structural and infrastructural barriers compound the cognitive and logistical burdens of digital engagement [10]. Expensive or unreliable mobile data, limited phone storage, reliance on shared devices, and varying digital literacy make it even harder for users to manage multiple tools effectively. These technical barriers are further reinforced by weak governance structures, short-term donor initiatives, and poor integration with national health systems, limiting sustainability and risking deeper inequities [11].

In the sections that follow, we discuss two complementary strategies for addressing app fatigue and advancing more sustainable digital health ecosystems: first, managing fragmentation through greater platform consolidation and integration across clinical and public health systems; and second, moving beyond isolated app development toward embedding digital health functions within the familiar platforms and social networks people already use and rely upon.

## Managing fragmentation: The role of developers and system stakeholders

One promising approach consolidates functions into fewer, comprehensive platforms through “gateway” platforms, open standards, and personalization [4]. Integrated platforms offer clear advantages: stronger oversight, improved data governance, consistent and improved user experiences, and reduced duplication. Real-world examples illustrate some of these benefits: an Irish Adult ADHD App provided a single, evidence-based source of psychoeducation, attracting over 12,000 active users in its first year [12]. Similarly, the Agatha prototype—a comprehensive digital healthy aging coach developed in partnership with the World Health Organization—demonstrated how consolidation advances equity by reaching older adults through participatory design emphasizing simple navigation and voice activation [13].

However, drawbacks remain. Even consolidated platforms require learning new tools, a major burden for those with limited digital literacy. As features accumulate, one-size-fits-all designs can be slow to adapt across contexts. The NHS App illustrates these tensions: while scaling to millions of users and improving access to prescriptions and records, evaluations revealed uneven uptake across age, literacy, and socioeconomic groups, with inconsistent feature rollouts leaving patients with uneven access [14]. However, complete consolidation is often unrealistic and sometimes undesirable. Different users and contexts require flexibility. Health systems can help by guiding patients toward curated sets of evidence-based, interoperable apps, reducing confusion while preserving flexibility. To make such guidance actionable, enterprise architecture on the back end can connect mobile apps with clinical and laboratory systems, enabling secure data exchange, coordinated workflows, and clearer governance across platforms [15]. Taken together, these system supports enable learning health systems in which usage and outcome data feed continuous improvement [16].

Realizing this vision also requires aligning developer incentives with system goals, particularly in commercial app ecosystems. Current compensation models often reward scale over outcomes, fueling engagement-optimized rather than impact-focused development. Performance-based payment models linking rewards to measurable health improvements (lower blood pressure, better medication adherence, improved well-being) offer a path forward. By incentivizing patient outcomes over reach, such models can transform fragmentation into an asset.

## Beyond better apps: Embedding digital health in everyday life

To truly address app fatigue, digital health must move beyond building better apps toward embedding interventions in platforms that are part of everyday life. This aligns with existing habits and trust, lowers onboarding friction, and expands access for those most likely to be left behind. Messaging platforms illustrate this potential. WhatsApp, ubiquitous across Asia, Africa, and Latin America, has been widely used for provider communication, patient education, and public health campaigns, particularly in low- and middle-income countries where WhatsApp’s low cost and familiarity support sustained engagement [17]. For example, an Indian hospital system launched a free WhatsApp channel providing diabetes education within a platform patients already use daily [18]. Similar roles are played by WeChat, LINE, and Telegram in other regions. The equity case is strong. Older adults with high chronic disease burdens and populations using simpler phones still routinely use platforms like WhatsApp to stay connected. Embedding digital health in these spaces extends reach through existing networks while requiring fewer resources than developing standalone apps, critical for scaling in low- and middle-income countries.

Likewise, recent evidence shows that mHealth apps often rely on overly simplistic, individualized designs that fail to reflect the complex aspects of human interaction and networked care [8]. Many of these approaches are rooted in “quantified self” frameworks, which position self-tracking and numerical optimization as a key means of managing and improving health, reducing the social and relational dimensions of health to decontextualized metrics that prioritize individual responsibility over collective agency [19]. Addressing these gaps requires moving beyond access to empowerment models, using digital platforms to equip individuals and communities as change agents. Peer support models demonstrate how this reduces inequities by fostering trust, strengthening social ties, and creating culturally relevant pathways [20]. Digital peer support has benefited older adults by building confidence, sustaining engagement, and reducing isolation [21]. Moreover, young adults can also act as conduits into family systems; the Let’s Chat intervention trained Vietnamese American young adults to spark cancer prevention conversations in family group chats, prompting preventive action among otherwise hard-to-reach older relatives [22]. These examples show that meeting people where they are digitally means integrating interventions in trusted platforms and empowering peers to drive diffusion.

Nonetheless, embedding health interventions in third-party platforms raises privacy, consent, and oversight concerns, especially where governance is weak [23]. A strategic approach uses platforms like WhatsApp for outreach, education, reminders, and support while reserving sensitive tasks (storing records, transmitting results) for secure clinical systems. This requires clear consent flows, minimal data collection, and transparent communication about data use. This layered model enhances accessibility, reduces app burden, and allows preferred-level engagement while promoting equity and ethical implementation. A diabetes patient might receive reminders via WhatsApp while accessing results through a hospital portal, making digital health more accessible and sustainable while preserving trust.

## Moving forward

App fatigue compels rethinking innovation—not simply new apps, but new ways of embedding health into people’s digital and social ecosystems. The shift must be from novelty to familiarity, proliferation to integration, and reach to meaningful, equitable impact. Sustainable digital health will be built on trusted experiences that reduce cognitive burden, align with routines, and strengthen rather than strain health systems. Advancing this vision will also require a stronger evidence base on app fatigue (including its prevalence, drivers, and effects on trust and sustained engagement) to inform designs that minimize burden and promote long-term use. Equity must remain central. Without attention to literacy, access, and cultural context, new tools risk reinforcing the gaps they aim to close. By reframing innovation around familiarity, trust, and equity, digital health can evolve from a fragmented landscape into an inclusive, resilient part of health systems. If achieved, the long-term promise of digital health (better outcomes, stronger systems, and healthier lives) remains within reach.
